# Supplementary material for: Spatiotemporal mapping of the contractile and adhesive forces sculpting early C. elegans embryos
Source: bioRxiv. 2025 Jul 1:2023.03.07.531437. Preprint. [Version 3] doi: 10.1101/2023.03.07.531437 (PMC12236823; doi:10.1101/2023.03.07.531437)
Supplement: Supplement 1 [file NIHPP2023.03.07.531437v3-supplement-1.pdf]

bioRxiv preprint doi: https://doi.org/10.1101/2023.03.07.531437; this version posted July 1, 2025. The copyright holder for this preprint (which was not certified by peer review) is the author/funder, who has granted bioRxiv a license to display the preprint in perpetuity. It is made available under aCC-BY 4.0 International license.

Supplemental figures

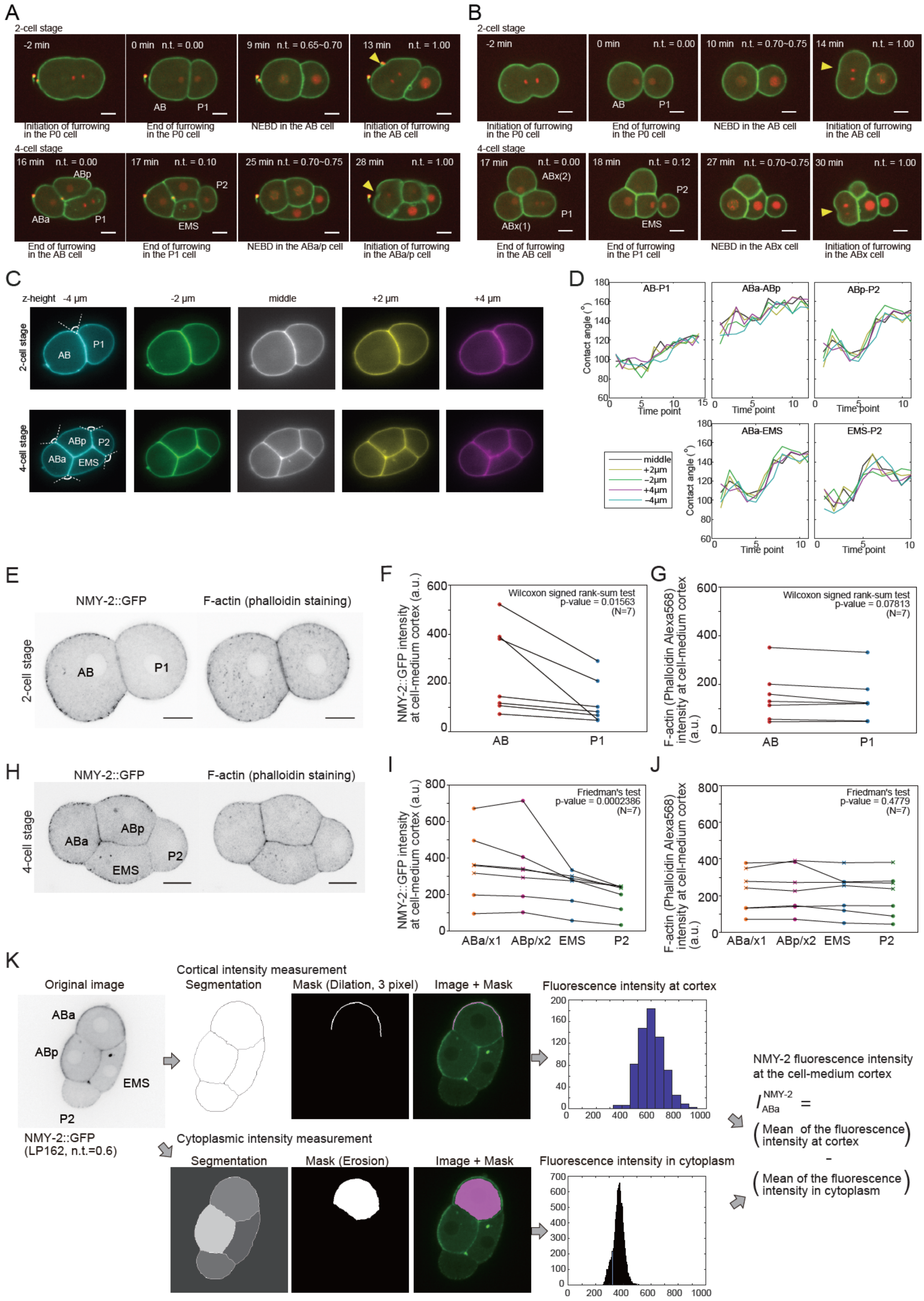

bioRxiv preprint doi: https://doi.org/10.1101/2023.03.07.531437; this version posted July 1, 2025. The copyright holder for this preprint (which was not certified by peer review) is the author/funder, who has granted bioRxiv a license to display the preprint in perpetuity. It is made available under aCC-BY 4.0 International license.

Figure S1: **(A-B)** Representative evolution of the cell arrangement in 2- and 4-cell *C. elegans* embryos. Cell membranes are visualized using GFP-PH-PLCδ and histones are visualized using mCherry::his-58. Scale bars =10µm. Yellow arrowheads indicate the position of furrowing. Cell names are indicated on each panel. Actual times are indicated in the top right corner. Normalised times (n.t.) are indicated in the top right corner. For the 2-cell stage, n.t.=0 was chosen as the time when P_0 finishes its division and for the 4 cell-stage, it was chosen as the time when AB finishes its division. For the 2-cell stage, n.t.=1 was chosen as the time when AB starts to furrow and, for the 4-cell stage, it was chosen as the moment when ABa/p/x begins to furrow. **A**. Representative evolution of cell arrangement for *C. elegans* embryos in the eggshell. **B**. Representative evolution of cell arrangements for *C. elegans* embryo outside of the eggshell. **C**. Consecutive images of a confocal microscopy stack taken at different optical planes. The position of each plane is given relative to the mid-plane of the embryo. Top row: 2-cell stage. Bottom row: 4-cell stage. The name of each cell is indicated on the left most image. The angles of contact are displayed in the leftmost column. **D**. Temporal evolution of the angles of contact shown in C. Each line represents a different optical plane. Angular evolution is not affected by small differences in the optical plane chosen for measurement. **E**. NMY-2::GFP and F-actin distribution in the midplane of 2-cell stage *C elegans* embryos. The name of each cell is indicated on the images. Scale bar = 10µm. **F**. NMY-2 fluorescence intensities measured in the cortex of each cell. Data from 7 embryos, each embryo appears as a separate dot. Cells from the same embryo are linked by black lines. Fluorescence intensities were compared with a signed Wilcoxon rank-sum test. **G**. F-actin fluorescence intensities measured in the cortex of each cell. Data from 7 embryos, each embryo appears as a separate dot. Cells from the same embryo are linked by black lines. Fluorescence intensities were compared with a signed Wilcoxon rank-sum test. **H**. NMY-2::GFP and F-actin distribution in the midplane of 4-cell stage *C elegans* embryos. The name of each cell is indicated on the images. Scale bar = 10µm. **I.** Same as F but for the 4-cell stage. Fluorescence intensities were compared with a Friedman's test. **J**. Same as G but for the 4-cell stage. Fluorescence intensities were compared with a Friedman's test. **K**. Image analysis pipeline for extracting fluorescence intensities at interfaces. This is exemplified on NMY-2::GFP at the 4-cell stage. Top row shows cortical intensity measurement and the bottom row the cytoplasmic intensity measurement. For the cortical intensity, cell surfaces are segmented using Tissue analyzer. Then the mask for a specific surface (here ABa) is dilated to have a 3 pixel width. Then, it is convolved with the image to give a histogram of fluorescence intensities. For the cytoplasmic intensity, a mask of the cytoplasm is generated and eroded. This is then convolved with the image to generated a histogram of fluorescence intensities. Note that fluorescence intensities in the cortex are larger than in the cytoplasm. The NMY-2 fluorescence intensity at the cell-medium cortex is then computed as the difference between the means of the fluorescence at the cortex and the cytoplasmic intensity.

bioRxiv preprint doi: https://doi.org/10.1101/2023.03.07.531437; this version posted July 1, 2025. The copyright holder for this preprint (which was not certified by peer review) is the author/funder, who has granted bioRxiv a license to display the preprint in perpetuity. It is made available under aCC-BY 4.0 International license.

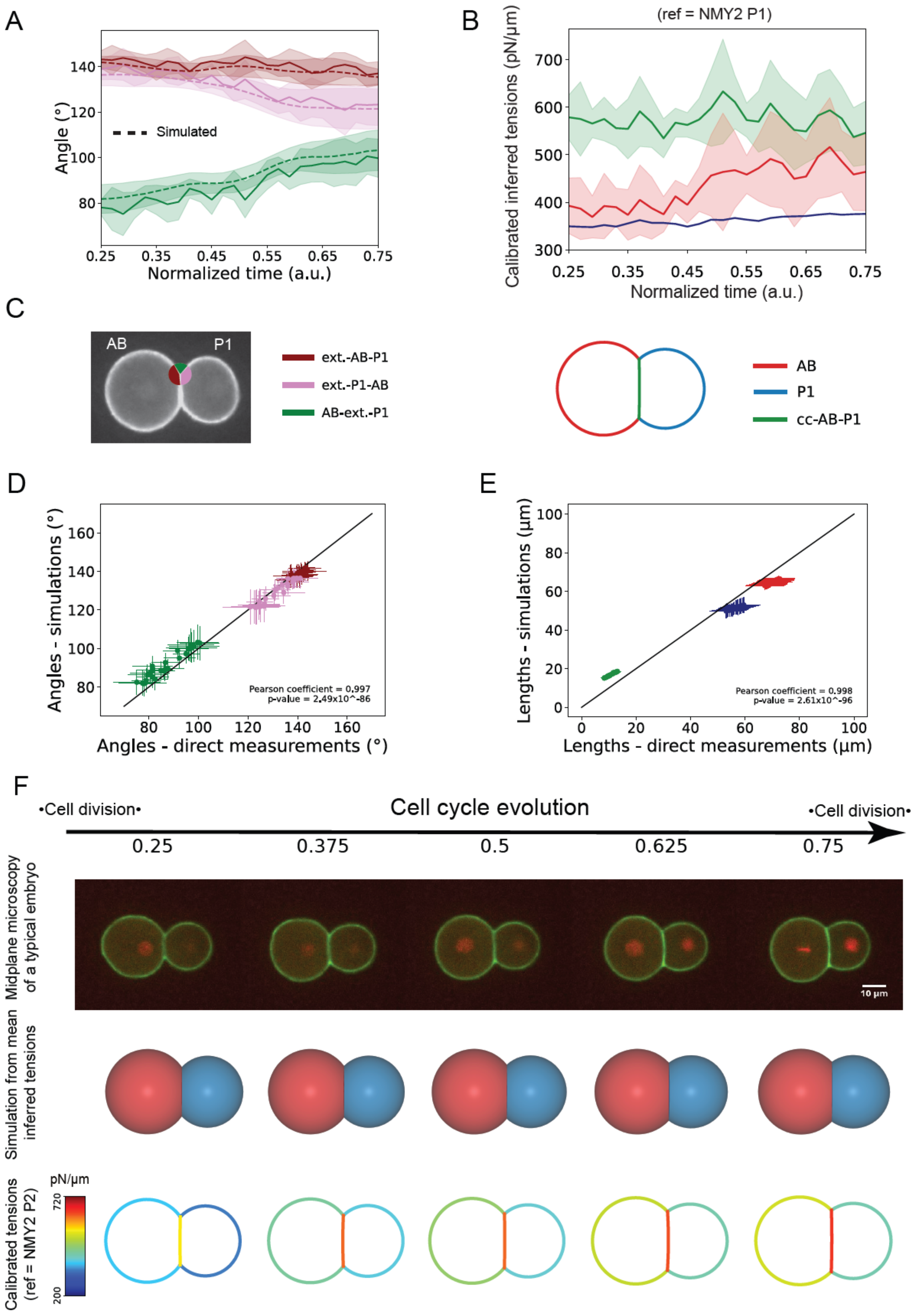

bioRxiv preprint doi: https://doi.org/10.1101/2023.03.07.531437; this version posted July 1, 2025. The copyright holder for this preprint (which was not certified by peer review) is the author/funder, who has granted bioRxiv a license to display the preprint in perpetuity. It is made available under aCC-BY 4.0 International license.

Figure S2: **Inference and simulation of the 2-cell stage embryo outside of the eggshell**. **A**. Temporal evolution of the external angles of contact at tricellular junctions. The solid line shows the mean and the shaded area represents the standard deviation (n=5 embryos). Time is normalised to the cell cycle and the color codes relating to external angles of contact are indicated in the panel C below. **B**. Temporal evolution of the mean inferred surface tensions calibrated in time and absolute value with the myosin fluorescence intensity in P1 and the affine parameters α=0.751 pN/(µm.ua), β=325 pN/µm. The solid line shows the average over 5 embryos and the shaded area represents the standard deviation. Time is normalised to the cell cycle. **C**. Left: Color code for contact angles in the 2-cell stage embryo used throughout the manuscript. Right: Color code for interfaces in the 2-cell stage embryo used throughout the manuscript. **D**. Plot of the contact angles obtained from simulations as a function of experimentally measured angles. Each data point represents one time point with standard deviation and is averaged over 5 embryos. The correlation is measured through a Pearson coefficient ρ=0.997. The black line shows the line of slope 1. **E.** Plot of the simulated lengths as function of experimentally measured lengths. Each data point represents one time point with standard deviation and is averaged over 5 embryos. The correlation is measured through a Pearson coefficient ρ=0.998. The black line shows the line of slope 1. **F.** Temporal evolution of inferred surface tensions allows prediction of the cell arrangement in embryos. First row: microscopy time series of a developing 2-cell stage embryo outside of the eggshell. The membrane is visualized with GFP-PH-PLCδ (green) and the histones are visualized with mCherry::his-58 (red). Scale bar=10µm. Second row: temporal evolution of the mean 3D embryo shape predicted by simulation (n=5 embryos). AB appears in orange and P_1 in blue. Third row: temporal changes in surface tension in pN/µm. The tension in each surface is color coded with blue representing low tension and red high tension.

bioRxiv preprint doi: https://doi.org/10.1101/2023.03.07.531437; this version posted July 1, 2025. The copyright holder for this preprint (which was not certified by peer review) is the author/funder, who has granted bioRxiv a license to display the preprint in perpetuity. It is made available under aCC-BY 4.0 International license.

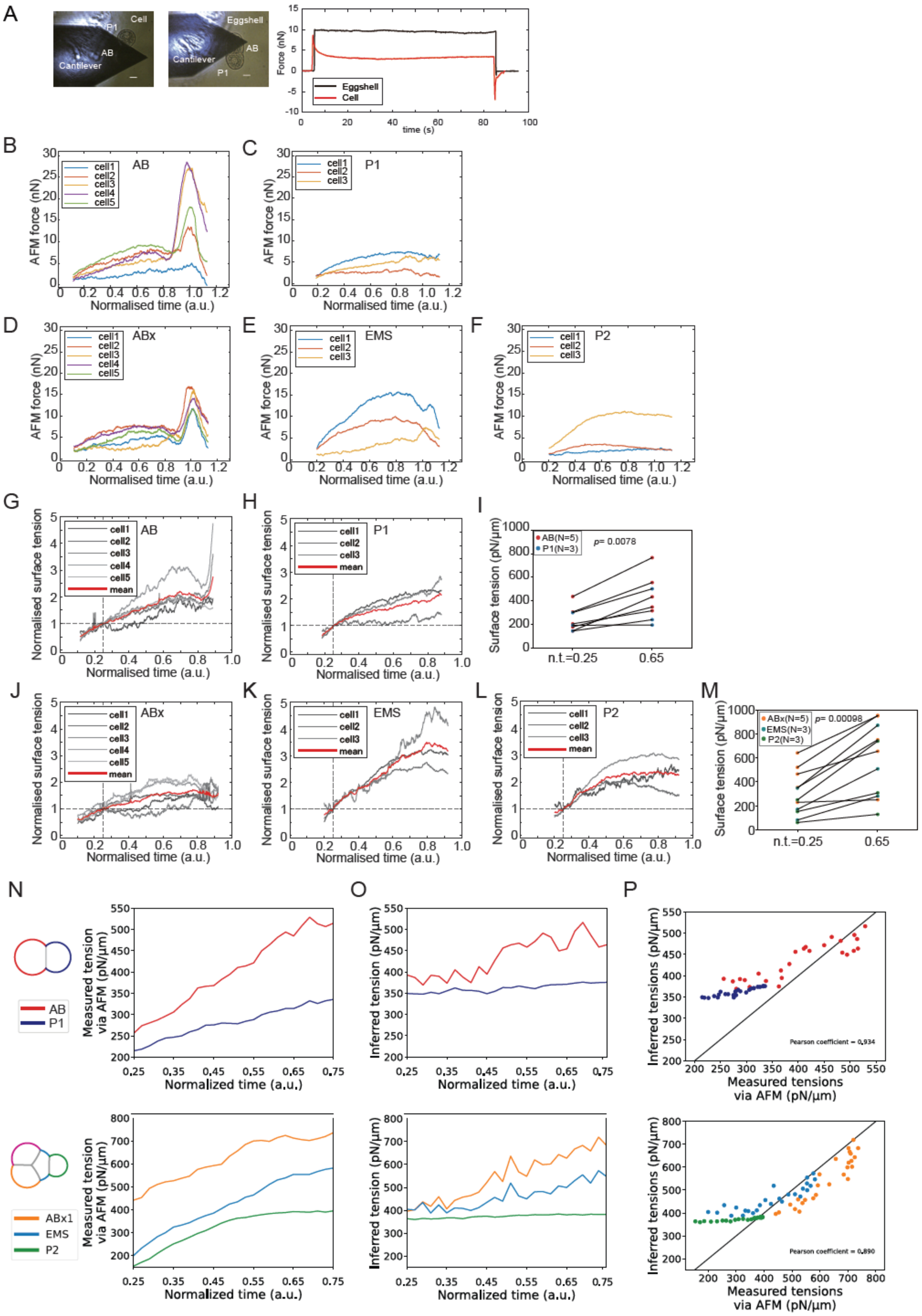

bioRxiv preprint doi: https://doi.org/10.1101/2023.03.07.531437; this version posted July 1, 2025. The copyright holder for this preprint (which was not certified by peer review) is the author/funder, who has granted bioRxiv a license to display the preprint in perpetuity. It is made available under aCC-BY 4.0 International license.

Figure S3: AFM measurements of cell surface tension. **A**. Representative AFM measurement of the mechanics of a cell and the eggshell. Left and middle: representative brightfield images of an AFM cantilever in contact with a 2-cell stage embryo without (left) and with (middle) an eggshell. The name of each cell is indicated on the images. Scale bar=10μm. Right: Representative graph showing the temporal evolution of the deflection force sensed by the AFM cantilever for a cell in an embryo without an eggshell (orange) or for the eggshell (blue). The cantilever is initially out of contact and senses no force. It is then brought into contact with the cell or the eggshell at t~5s, leading to an increase in the measured force. The cantilever is then kept at a constant height for 80s before being retracted, leading to a return of the force to 0. While the cantilever is in contact, the force measured on the cell decreases due to viscoelastic relaxation stemming from biological processes such as cytoskeletal turnover. In contrast, no such relaxation is observed on the eggshell pointing to a solid-like behavior. **(B-F)** Temporal evolution of force measured by AFM when in contact with AB cells (B), P_1 cells (C), ABx cells (D), EMS cells (E), and P_2 cells (F). In all graphs the unit of force is in 10^{-9} N. Each line represents a different cell. Measurements are acquired at 5kHz and smoothed with a moving window of 1s. **(G-H, J-L)** Temporal evolution of cell-medium cortical tension normalised to the tension at n.t.=0.25 for AB cells (G), P_1 cells (H), ABx cells (J), EMS cells (K), and P_2 cells (L). Each grey line represents a different cell and the mean is plotted as a red line. (**I, M**) Tension in cells at n.t.=0.25 and n.t.=0.65. Lines link data points corresponding to the same cell. A Wilcoxon signed rank-sum test was used to compare tensions between time points. **N**. Temporal evolution of cell-medium cortical tensions measured by AFM for the 2-cell (top) and 4-cell (bottom) embryos outside of their eggshell. Each cell-medium interface is colour-coded as shown on the sketches to the left. **O**. Temporal evolution of cell-medium cortical tensions predicted by our myosin-informed inference approach for the 2-cell (top) and 4-cell (bottom) embryos outside of their eggshell. Colour codes are the same as in L. **P**. Inferred cell-medium cortical tension as a function of the cortical tension measured by AFM for the 2-cell (top) and 4-cell (bottom) embryos outside of their eggshell. Colour codes are the same as in L. (**N-O**) AFM data is from the cells in B-F and fluorescence data is averaged over 5 embryos.

bioRxiv preprint doi: https://doi.org/10.1101/2023.03.07.531437; this version posted July 1, 2025. The copyright holder for this preprint (which was not certified by peer review) is the author/funder, who has granted bioRxiv a license to display the preprint in perpetuity. It is made available under aCC-BY 4.0 International license.

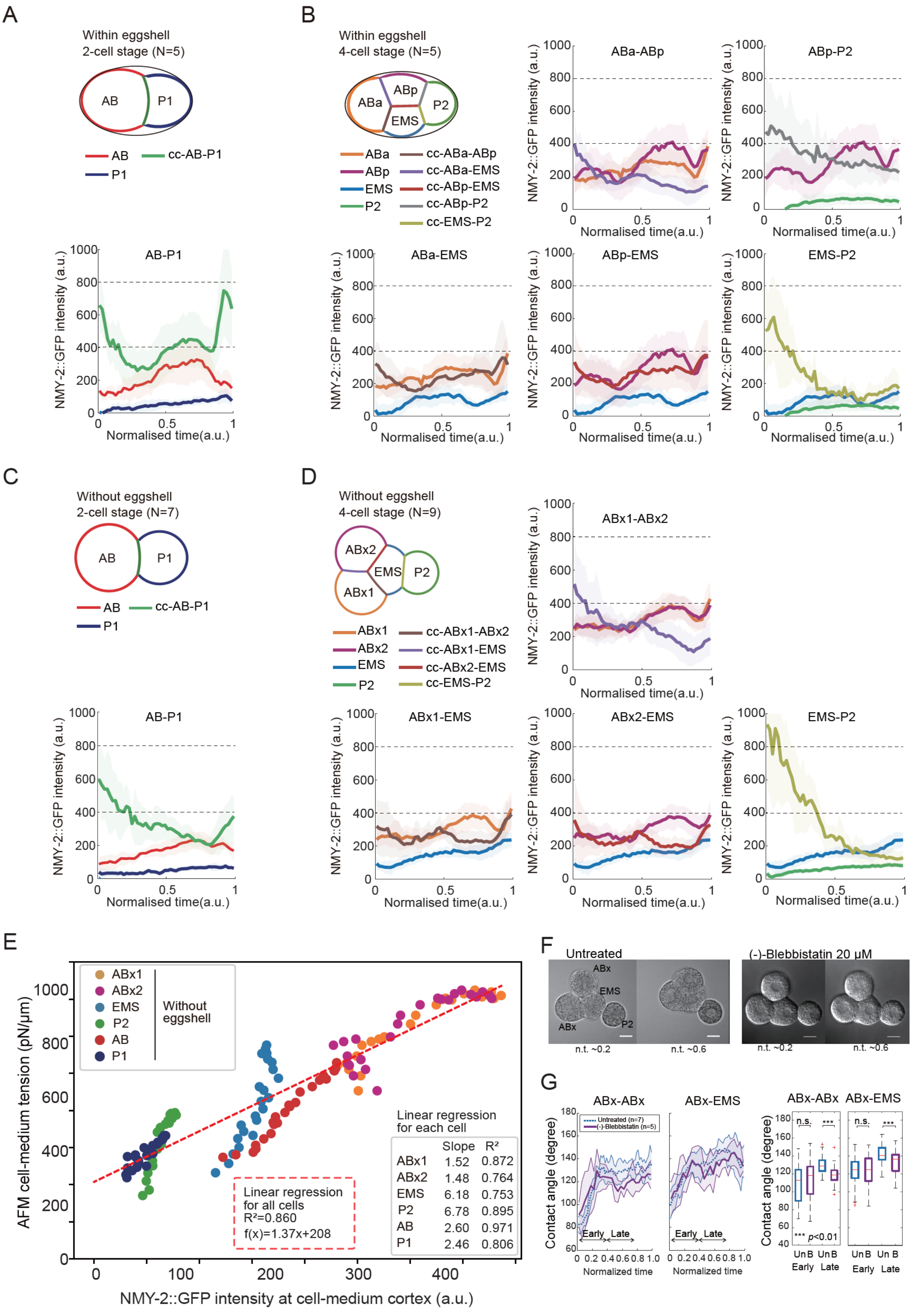

bioRxiv preprint doi: https://doi.org/10.1101/2023.03.07.531437; this version posted July 1, 2025. The copyright holder for this preprint (which was not certified by peer review) is the author/funder, who has granted bioRxiv a license to display the preprint in perpetuity. It is made available under aCC-BY 4.0 International license.

Figure S4 related to Figure 4: Temporal evolution of NMY-2 in the surfaces of the 2- and 4-cell *C. elegans* embryos with and without eggshell. (A-D) The pictograms indicate the color code for the surfaces of the embryos. In all graphs, the solid line is the average and the shaded region depicts the standard deviation. N≥5 embryos for each condition. Each graph shows the evolution of the fluorescence intensity of myosin in an intercellular contact (indicated as cc) and in the cortices on either side of that contact. All intensities are given in a.u. **A.** Temporal evolution of NMY-2 fluorescence intensity in 2-cell stage embryos within the eggshell. **B.** Temporal evolution of NMY-2 fluorescence intensity in 4-cell stage embryos within the eggshell. **C.** Temporal evolution of NMY-2 fluorescence intensity in 2-cell stage embryos outside of the eggshell. **D.** Temporal evolution of NMY-2 fluorescence intensity in 4-cell stage embryos outside of the eggshell. **E**. Cortical tension measured by AFM is plotted as a function of cell–medium myosin fluorescence intensity. Each data point corresponds to a single time point and represents the average over 5 embryos, with standard deviation indicated. The dashed red line indicates the best linear regression to the data from all cells (R^2=0.86), with slope and intercepts α=1.37 pN/(µm.ua), β=208 pN/µm. The slopes of the regression for each cell type are given in inset. **F**. Bright field images of 4-cell stage *C. elegans* embryo without an eggshell untreated (left) and blebbistatin treated (right) at n.t.=0.2 and 0.6. Scale bar 10 µm. **G**. Left : Temporal evolution of contact angles between ABx-ABx and ABx-EMS in untreated (blue) and blebbistatin treated conditions (purple). Right : Comparison of time-averaged contact angles between ABx-ABx and ABx-EMS in untreated (blue, “Un”) and blebbistatin treated condition (purple, “B”) at early (n.t.= from 0 to 0.4) and late (n.t.= from 0.4 to 0.8) stages. Contact angles were compared with a Wilcoxon rank-sum test.

bioRxiv preprint doi: https://doi.org/10.1101/2023.03.07.531437; this version posted July 1, 2025. The copyright holder for this preprint (which was not certified by peer review) is the author/funder, who has granted bioRxiv a license to display the preprint in perpetuity. It is made available under aCC-BY 4.0 International license.

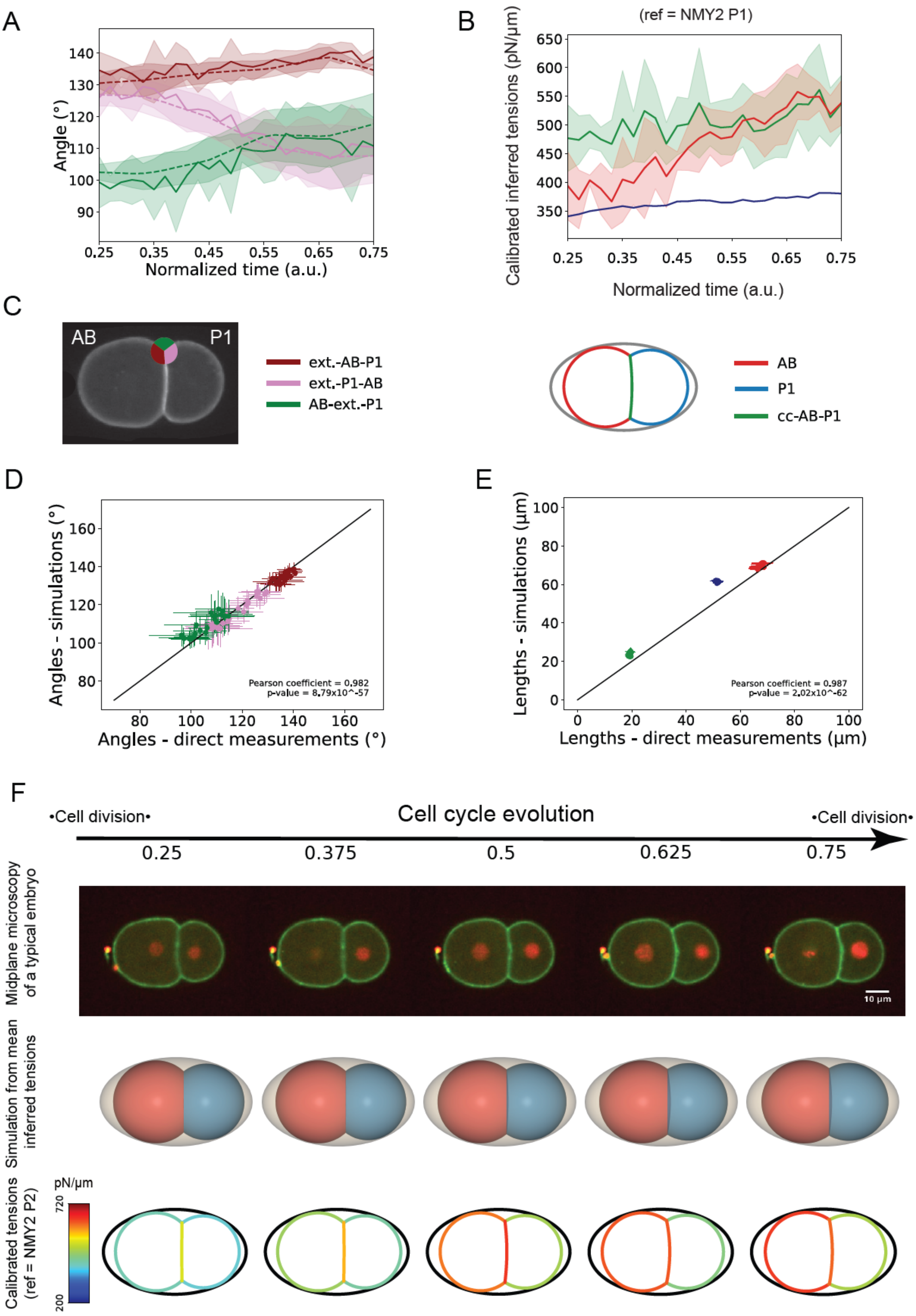

bioRxiv preprint doi: https://doi.org/10.1101/2023.03.07.531437; this version posted July 1, 2025. The copyright holder for this preprint (which was not certified by peer review) is the author/funder, who has granted bioRxiv a license to display the preprint in perpetuity. It is made available under aCC-BY 4.0 International license.

Figure S5: **Simulation of the 2-cell stage embryo inside the eggshell**. **A**. Temporal evolution of the external angles of contact at tricellular junctions. The solid line shows the mean and the shaded area represents the standard deviation (n=5 embryos). Time is normalised to the cell cycle and the color codes relating to external angles of contact are indicated in the panel C below. **B**. Temporal evolution of the mean inferred surface tensions calibrated in time and absolute value with the myosin fluorescence intensity in P1 and the affine parameters α=0.751 pN/(µm.ua), β=325 pN/µm. The solid line shows the average over 5 embryos and the shaded area represents the standard deviation. Time is normalised to the cell cycle. **C**. Left: Color code for contact angles in the 2-cell stage embryo used throughout the manuscript. Right: Color code for interfaces in the 2-cell stage embryo used throughout the manuscript. **D**. Plot of the contact angles obtained from simulations as a function of experimentally measured angles. Each data point represents one time point with standard deviation and is averaged over 5 embryos. The correlation is measured through a Pearson coefficient ρ=0.982. The black line shows the line of slope 1. **E.** Plot of the simulated lengths as function of experimentally measured lengths. Each data point represents one time point with standard deviation and is averaged over 5 embryos. The correlation is measured through a Pearson coefficient ρ=0.987. The black line shows the line of slope 1. **F.** Temporal evolution of inferred surface tensions allows prediction of the cell arrangement in embryos. First row: microscopy time series of a developing 2-cell stage embryo outside of the eggshell. The membrane is visualized with GFP-PH-PLCδ (green) and the histones are visualized with mCherry::his-58 (red). Scale bar=10µm. Second row: temporal evolution of the mean 3D embryo shape predicted by simulation (n=5 embryos). AB appears in orange and P_1 in blue. The shell is indicated in grey. Third row: temporal changes in surface tension in pN/µm. The tension in each surface is color coded with blue representing low tension and red high tension. The shell is indicated in black.

bioRxiv preprint doi: https://doi.org/10.1101/2023.03.07.531437; this version posted July 1, 2025. The copyright holder for this preprint (which was not certified by peer review) is the author/funder, who has granted bioRxiv a license to display the preprint in perpetuity. It is made available under aCC-BY 4.0 International license.

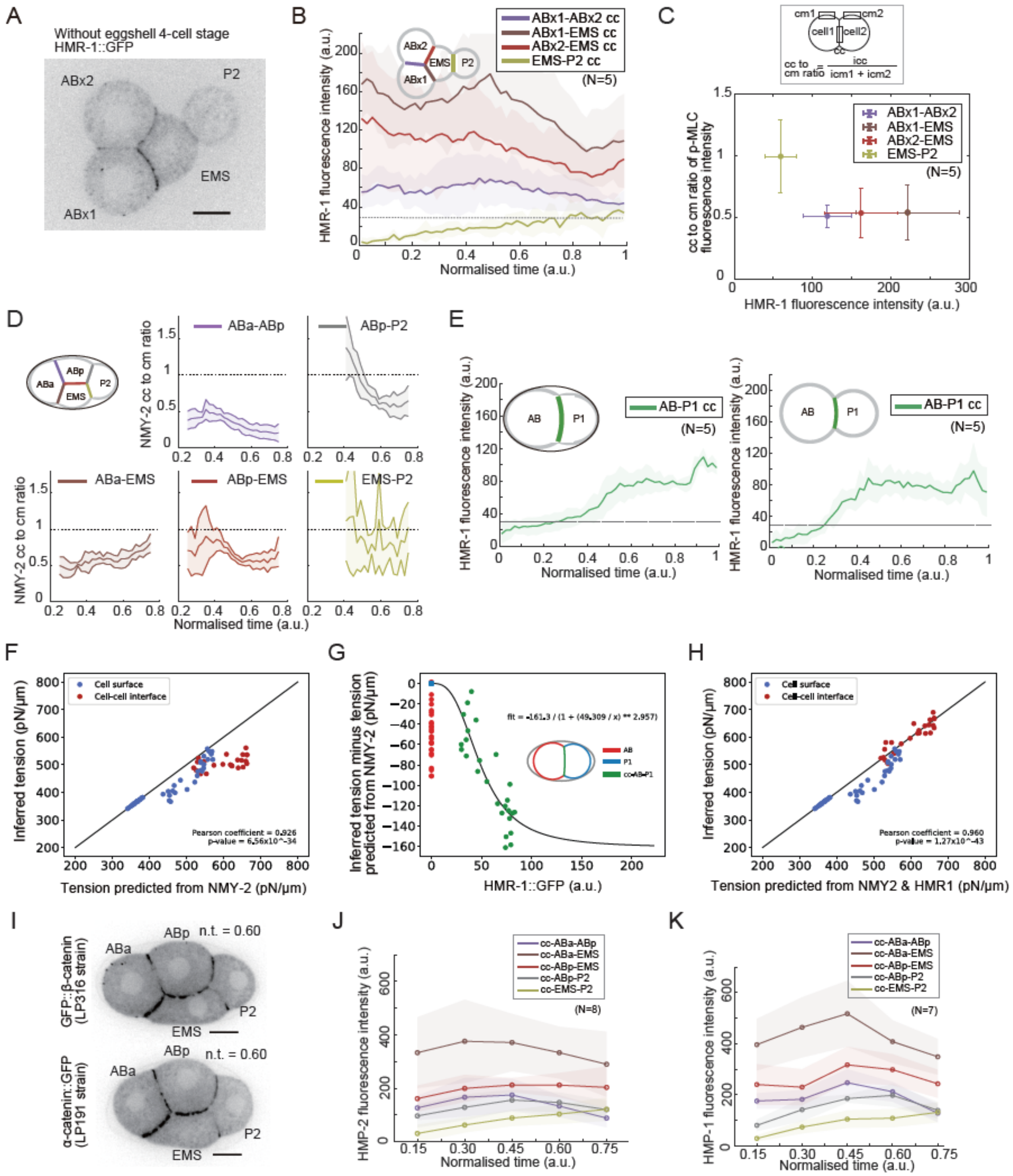

Figure S6: **Temporal evolution of HMR-1, α-catenin, β-catenin and myosin at cell-cell contacts in the *C. elegans* embryos.** A. E-cadherin-GFP (HMR-1) localization in a representative embryo at the 4-cell stage without the eggshell. Scale bar=10µm. The identity of each cell is indicated on the image. B. **B**. Temporal evolution of HMR-1 fluorescence intensity in 4-cell stage embryos without the eggshell. The inset pictogram indicates the position and colour-code of cell-cell contacts in the embryo. The solid line is the average and the shaded region depicts the standard deviation. N=5 embryos for each condition. The dashed line indicates the threshold fluorescence below which HMR-1 enrichment at cell-cell

bioRxiv preprint doi: https://doi.org/10.1101/2023.03.07.531437; this version posted July 1, 2025. The copyright holder for this preprint (which was not certified by peer review) is the author/funder, who has granted bioRxiv a license to display the preprint in perpetuity. It is made available under aCC-BY 4.0 International license.

contacts is not visually distinguishable from background. C. Top: sketch of the measurement of the ratio of p-MLC fluorescence intensity at intercellular contacts and at the cell-medium cortex in the two cells in contact. The p-MLC data is taken from Fig 4B-C. The ratio is computed as the intensity i_{cc} in the cell-cell contact divided by the sum of the intensities i_{cm1} and i_{cm2} in the cortices. Bottom: The ratio of myosin at cell-cell contacts to the myosin at cortices is plotted as a function of HMR-1 enrichment in each cell-cell contact (taken from the measurements in D, top panel is a representative image). Whiskers indicate the standard deviation. Data is averaged over 5 embryos. p-MLC decreases with increasing HMR-1 in the contact. D. Comparison between NMY-2::GFP fluorescence intensity at intercellular contacts and at the cell-medium cortex in each pair of cells in contact. The ratio is computed as the intensity I_{cc} in the cell-cell contact divided by the sum of the intensities I_{cm1} and I_{cm2} in the cortices. Data from 5 different embryos and 18 or 13 time points per embryo. **E. Left :** Temporal evolution of HMR-1 fluorescence intensity at the AB-P_1 contact for an embryo with an eggshell. Right : Temporal evolution of HMR-1 fluorescence intensity at the cell-cell contacts in the 4-cell stage embryo with an eggshell. **F**. Tensions inferred from angles as a function of tension predicted from myosin fluorescence intensity in 2-cell embryos within the eggshell. Tensions were inferred from myosin intensity using the relation $\gamma^{\mathrm{NMY2}} = \alpha.I^{\mathrm{NMY2}} + \beta$, with α=0.751 pN/(µm.ua), β=325 pN/µm. Cell-medium surfaces are plotted in blue and cell-cell contacts in red. Each point corresponds to a given interface and time point and is averaged over 5 embryos. The correlation is measured through a Pearson coefficient ρ=0.926. The solid black line indicates a perfect correlation. **G**. Residual tension $\gamma^{\mathrm{residual}} = \gamma^{\mathrm{infer}} - (\alpha.I^{\mathrm{NMY2}} + \beta)$ at cell-medium and cell-cell interfaces in 2-cell stage embryos within the eggshell as a function of E-cadherin fluorescence intensity (HMR-1::GFP). Each given cell-cell interface is attributed a different color. Each point corresponds to a given interface and time point and is averaged over 5 embryos. Cytoplasmic background fluorescence was removed from the HMR-1 signal. The fit corresponds to a Hill function of the form $\gamma^{\mathrm{residual}} = -L\frac{(I^{\mathrm{HMR1}})^n}{K^n+(I^{\mathrm{HMR1}})^n}$ with a Hill coefficient n=2.96**,** a constant K=49.31 a.u. and a scale L=161.3 pN/µm. **H**. Surface tension at cell-medium interface (blue dots) and cell-cell interfaces (red dots) as a function of tension predicted from myosin and E-cadherin fluorescence intensity in 2-cell stage embryos within the eggshell. Each point corresponds to a given interface and time point. Data is averaged over 5 embryos. The correlation is measured through a Pearson coefficient ρ=0.980. The solid black line indicates a perfect correlation. **I**. Top: Representative images of β-catenin (HMP-2) in the 4-cell *C. elegans* embryo with an eggshell. Bottom: Representative images of α-catenin (HMP-1) in the 4-cell *C. elegans* embryo with an eggshell. Scale bars=10µm. **J**. Temporal evolution HMP-2 fluorescence intensity at the cell-cell contacts in the 4-cell stage embryo with an eggshell. The solid line is the average and the shaded region depicts the standard deviation. Data from N=8 embryos. **K**. Temporal evolution HMP-1 fluorescence intensity at the cell-cell contacts in the 4-cell stage embryo with an eggshell. The solid line is the average and the shaded region depicts the standard deviation. Data from N=7 embryos.

bioRxiv preprint doi: https://doi.org/10.1101/2023.03.07.531437; this version posted July 1, 2025. The copyright holder for this preprint (which was not certified by peer review) is the author/funder, who has granted bioRxiv a license to display the preprint in perpetuity. It is made available under aCC-BY 4.0 International license.

Supplemental videos

Video S1. Time-lapse movie of a developing *C. elegans* embryo expressing a GFP-tagged membrane marker and mCherry-tagged histone marker and manually deprived of its eggshell from zygote stage. Embryos were observed from 1-cell to 2-cell stage, related to Figure S1, S3 (left), and from 2-cell to 4-cell stage, related to Figures 1, S1 (right). Scale bar = 10μm.

Video S2. Movies of 3D heterogeneous foam simulations of the *C. elegans* embryo without an eggshell at the 2-cell stage (left) and 4-cell stage (right), related to Figures 2, S2.

Video S3. Measurement of cortical tension dynamics using AFM, related to Figure 3, S3. The AFM cantilever was left in contact with the cell for the duration of its cell cycle.

Video S4. Time-lapse movie of a developing *C. elegans* embryo expressing a GFP-tagged membrane marker and mCherry-tagged histone marker within its native eggshell. Embryos were observed from 1-cell to 8-cell stage, related to Figures 2, 5, S1, S2, S4 (left). *C. elegans* embryo expressing a GFP fusion to the endogenous NMY-2 gene within the eggshell from 1-cell to 8-cell stage, related to Figures 4, S4 (right). Scale bar = 10μm.

Video S5. Movies of 3D heterogeneous foam simulations of the *C. elegans* embryo within the eggshell at the 2-cell stage (left) and 4-cell stage (right), related to Figures 5, S5.

Video S6. Time-lapse movie of a developing *C. elegans* embryo expressing a GFP fusion to the endogenous HMR-1 gene within the eggshell from 1-cell to 8-cell stage, related to Figures 6, S6. Scale bar = 10μm.
